# Investigation of Trimethylenemethane Cyclopentyl-Annulations as a Strategy to Obtain a Functionalized Angular Triquinane Skeleton †

**DOI:** 10.3390/molecules29225358

**Published:** 2024-11-14

**Authors:** S. Mason Webber, Wade A. Russu

**Affiliations:** Department of Pharmaceutical Sciences, Thomas J. Long School of Pharmacy, University of the Pacific, Stockton, CA 95211, USA; m_webber@u.pacific.edu

**Keywords:** angular triquinane, trimethylenemethane, TMM, diradical, bicyclo[3.3.0]octenones

## Abstract

The angular triquinane carbocyclic ring system is a component of many natural products found in numerous terrestrial and marine plants. A strategy for the synthesis of functionalized angular triquinanes utilizing two trimethylenemethane (TMM)-based [3+2] cycloaddition reactions is presented. This synthetic strategy employs the intermolecular dyil-trapping reaction to give eventual access to the bicyclo[3.3.0]oct-1-en-3-one system. A subsequent [3+2] cycloaddition with a TMM equivalent provides the angular triquinane carbocyclic framework.

## 1. Introduction

Triquinanes, or polycarbocyclic ring systems composed of three five-membered rings, occur in numerous terrestrial and marine plants. These naturally occurring quinanes have been the subject of synthetic and biological investigations for many decades [1,2]. The five-membered rings can be adjoined in a linear, angular, or propellane pattern. These triquinane molecules possess biological and medicinal value [3,4,5]. The family of angular triquinanes is characterized by three five-membered rings fused in an angular pattern so that all three rings share a common quaternary carbon in an all-cis-fused fashion. Sub-families of angular triquinanes are characterized by the position of the methyl groups on the carbocyclic skeleton. Specific angular triquinanes are characterized by their specific structure, including their stereochemistry, such as silphinene, pentalenene, and subergorgic acid. Natural angular triquinanes also serve as biosynthetic precursors to pentalenolactone antibiotics [6]. We are interested in angular triquinanes because we believe that synthetic access to unique derivatives has the potential to deliver new drug lead molecules. This belief leads us not toward the total synthesis of a particular natural product but to the development of a general synthetic methodology to angular triquinane-bearing functional groups readily usable for analog synthesis.

**Scheme 1:**
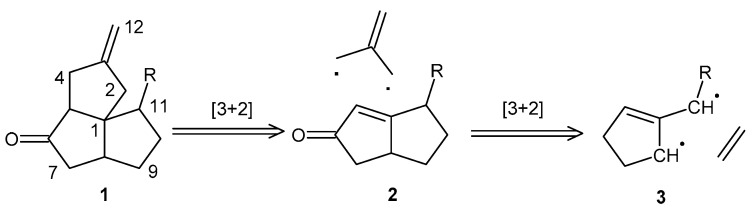
Retrosynthetic scheme depicting the TMM-based [3+2] cycloaddition strategy to access functionalized angular triquinanes.

We envisioned a relatively quick access to the angular triquinane ring system, with each ring bearing a synthetically useful functionalization, by the direct [3+2] cycloaddition of a trimethylenemethane (TMM) unit with a functionalized bicyclo[3.3.0]oct-1-en-3-one. This ring system can be accessed by the [3+2] addition of a TMM-based diradical with an olefin (Scheme 1) [7,8,9]. Reports in the literature of the direct cyclopentyl-annulation of bicyclo[3.3.0]oct-1-en-3-ones toward angular triquinanes are limited to two-step annulations using conjugate-addition reactions with sulfinylallyl anions or bifunctional cuprates, sometimes as one-pot reactions [10,11,12,13]. The drawbacks of using bifunctional reagents are additional synthetic reactions, the use of organotin reagents, and potentially sensitive transmetallation steps. There are also examples of Danheiser annulation with similar substrates having been successful in giving access to angular tetra- and triquinanes [14,15,16]. The drawback of employing this approach is the use of a strong Lewis acid that may not be tolerated by some functional groups. There are no examples of the direct cyclopentyl-annulation of bicyclo[3.3.0]oct-1-en-3-one with a TMM equivalent. We are aware of one other TMM-based diradical strategy toward angular triquinanes that involves the intramolecular [3+2] cycloaddition of a diazene-derived TMM in route to the total synthesis of crinipellin A [17,18,19]. Our motivation for investigating this synthetic route is the production of a useful intermediate for elaboration into non-natural angular triquinane analogs for the eventual identification of new antimicrobial and anticancer drug lead molecules.

## 2. Results and Discussion

**Scheme 2:**
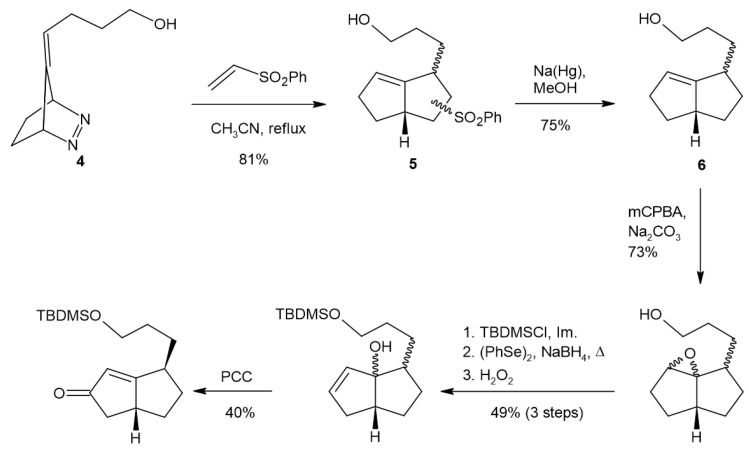
Synthesis of bicyclo[3.3.0]octeneone **9**.

To test our proposed route to the creation of functionalized angular triquinanes, we used diazene **4** (Scheme 2) [20,21]. This compound provides the functionalization of one of the rings of the product in the form of a tether that could be used for the future attachment of various labeling moieties. Heating **4** in degassed acetonitrile to generate the corresponding TMM diyl, in the presence of phenylvinylsulfone as the diylophile, resulted in the formation of **5** as a mixture of isomers. Phenylvinylsulfone was chosen as the diylophile because of its ability to serve as an ethylene and a ketene equivalent [22,23,24]. The phenylsulfone group was removed under reducing conditions with sodium amalgam to provide compound **6** and reveal relative stereoselectivity in the diyl-trapping reaction. Purification by column chromatography provided the major isomer contaminated with a small amount of the minor isomer. The nuclear magnetic resonance (NMR) spectra (^1^H, ^13^C, TOCSY, HMQC, NOSEY) were consistent with the syn-relative stereochemistry of the predominant isomer. The vinyl and ring juncture methine ^1^H chemical shifts in the major isomer of **6** appeared downfield of the same resonances for the minor isomer. Additionally, the ^13^C resonances of the ring juncture methine carbon and the ring juncture vinyl were downfield in the case of the major isomer, and the methinyl vinyl carbon was upfield compared to the minor isomer (Figures S4–S6). This type of stereoselectivity in the intermolecular diyl-trapping reaction has previously been observed [25,26,27,28]. Attempts at the allylic oxidation of TBDMS-protected **6** in order to access **9** using SeO_2_ or chromium oxide were unsuccessful. A multistep procedure through epoxide **7** and allylic alcohol **8** provided the desired enone. The epoxidation of **6** with *m*-chloroperbenzoic acid (mCPBA) exhibited a low facial selectivity and provided a mixture of diastereomers, presumably due to steric hinderance or hydrogen bonding effects of the tether moiety. The integration of the two epoxide methine proton resonances, present in the product ^1^H NMR spectra, indicated a 4:1 ratio of isomers. This result is consistent with the formation of both cis- and trans-fused products resulting from the epoxidation of similar systems [29]. Attempts to open the epoxide with a strong base to obtain allylic alcohol **8** were unsuccessful, but the phenylselenide ion facilitated nucleophilic opening of the epoxide, followed by oxidative elimination, producing allylic alcohol **8** [30,31]. Finally, oxidation of the allylic alcohols using pyridinum chlorochromate (PCC) provided the desired enone, **9** [32].

**Figure 1:**
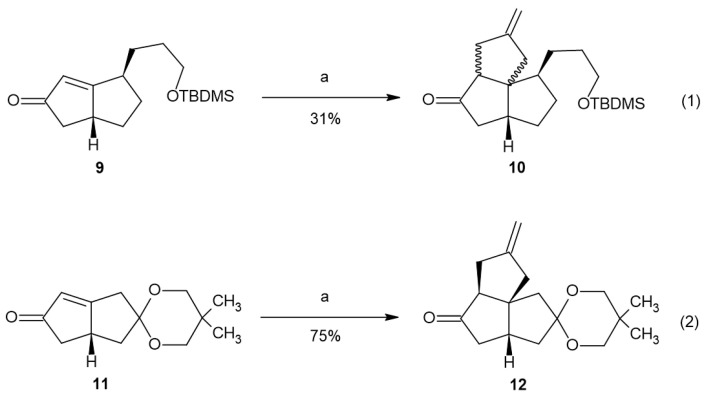
The [3+2] cycloaddition of **9** and **11**. Conditions: a—TMSCH_2_C(CH_2_)CH_2_OAc, Pd(Ph_3_P)_4_, dppe, THF, and reflux.

Enone **9** was subjected to [3+2] cycloaddition with Trost’s TMM precursor (Figure 1(1)) [33,34,35,36,37]. To our disappointment, a mixture of isomers was observed as the product. The expectation that the reaction of the bicyclo[3.3.0]oct-1-en-3-one system should exhibit facial selectivity to produce the thermodynamically more stable all-cis-fused angular triquinane product was not realized. The mixture of isomers consisting of the expected all-cis-fused product and the mixed cis–trans-fused product was not readily separable. Mass spectrometry confirmed the presence of **10**. The ^1^H NMR of the product mixture showed two distinct pairs of methylene vinyl peaks in a 60:40 ratio, estimated by integration (Figure S17). This result is consistent with our initial assignment of the relative stereochemistry of a major isomer of **6** and its subsequent epoxidation result. We believe that the tether present in the allylic position of molecule **9** presents a significant steric effect to the approach of the palladium–TMM complex.

**Figure 2:**
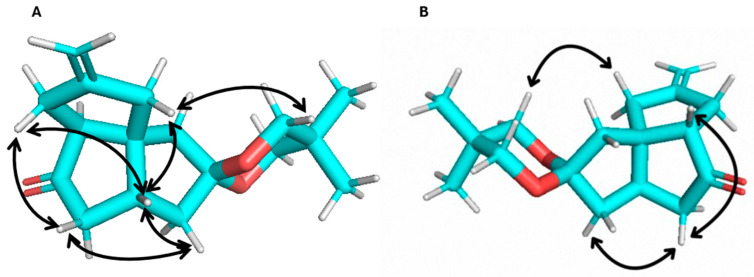
Observed NOEs for compound **12**: (**A**) β-face and (**B**) α-face views.

To test whether the tether present in the allylic position was the cause of the creation of the isomeric mixture in the [3+2] cycloaddition reaction, we subjected enone **11** to the same reaction conditions. Only one major isomer, **12**, was isolated from the reaction (Figure 1(2)). It is likely that another isomer was also formed. The NMR (^1^H, ^13^C, TOCSY, NOESY) data support product **12** as the all-cis-fused angular triquinane. The NMR chemical shifts were consistent with similar angular triquinanes [13,38]. Notable nuclear Overhauser effects (NOEs) were observed between C2Hβ and C8H and between the ketal methylene protons and both C2Hα and C5H (Figure 2). It should be noted here that **12** possesses a carbonyl or carbonyl equivalent in each of the cyclopentane rings, thus having the synthetic potential to further functionalize all but the quaternary carbon atom.

## 3. Conclusions

In conclusion, we have demonstrated that the intermolecular trapping of a diazene-derived TMM-based diyl may be used in route, via [3+2] cycloaddition with an olefin, to the bicyclo[3.3.0]oct-1-en-3-one system. The subsequent [3+2] cyclopentyl annulation of this ring system with a TMM equivalent can give access to the angular triquinane carbocyclic framework. The synthesis of **9** was plagued by the persistence of a minor isomer and low facial selectivity in the epoxidation step. Low facial selectivity was again observed in the [3+2] cycloaddition of **9** to give a mixture of two major isomers. To test the hypothesis that the allylic substituent in **9** was the cause of the formation of significant amounts of the undesired isomer, **11** was subjected to the reaction conditions. The reaction formed one major isomer of **12**, and this result supports the hypothesis. The advantage of using this TMM equivalent strategy is that the TMM precursor is commercially available and provides a masked carbonyl in the product. The route presented here complements previous approaches but possesses the drawback of sensitivity to allylic substitution in the bicyclo[3.3.0]oct-1-en-3-one substrate, which reduces facial selectivity, resulting in the formation of significant amounts of undesired isomers.

## Data Availability

No new data were created or analyzed in this study. Data sharing is not applicable to this article.

## Supplementary Materials

The following supporting information can be downloaded at: https://www.mdpi.com/article/10.3390/molecules29225358/s1, Experimental procedures and compound characterization data.
