# Supplementary material for: Investigation of Trimethylenemethane Cyclopentyl-Annulations as a Strategy to Obtain a Functionalized Angular Triquinane Skeleton
Source: Molecules. 2024 Nov 14;29(22):5358. doi: 10.3390/molecules29225358 (PMC11596830; doi:10.3390/molecules29225358)
Supplement: Supplementary file 1 [file molecules-29-05358-s001.zip › molecules-3269593-supplementary.pdf]

Supplementary Materials

Experimental

General. All reagents, solvents and starting materials were purchased and used without further purification unless otherwise indicated. Reactions were monitored by thin layer chromatography (TLC) on precoated silica gel F254 plates (EMD). TLC plates were visualized with UV light or p-anisaldehyde stain. Column chromatography was performed with EMD 230-400 mesh silica gel 60 Å. Yields are reported for isolated compounds. Melting points were determined using a Thomas Hoover apparatus and are uncorrected. ^{1}H and ^{13}C NMR spectra were recorded on a JEOL JNM-ECA600 spectrometer. Mass spectra were collected using an API3000 instrument. High resolution mass spectra for accurate mass determination were collected using a Waters GTC Premier TOF instrument.

3-[3-(Phenylsulfonyl)-1,2,3,3a,4,5-hexahydropentalen-1-yl]propan-1-ol and 3-[2-(Phenylsulfonyl)-1,2,3,3a,4,5-hexahydropentalen-1-yl]propan-1-ol (**5**):

Diazene **4** (0.97 g, 5.8 mmol) was dissolved in 116 mL of acetonitrile in a 250 ml round-bottom flask. The flask was plugged with a rubber stopper and an exit needle and degassed with nitrogen gas for approximately 15 minutes while stirring. To this flask was then quickly added 2.9 g (17.4 mmol) of phenyl vinyl sulfone all at once. The reaction mixture was then attached to a reflux condenser, sealed with Teflon tape, placed under nitrogen gas, and then put onto an oil bath at a temperature of 90 °C. Reaction was refluxed and complete after 2.5 hours at which point it was removed from the oil bath and allowed to cool down to rt. Workup was not necessary. Excess phenyl vinyl sulfone was crystalized out of solution with isopropyl alcohol and removed using a Buchner funnel filtration. The filtrate was collected, concentrated and purified using silica gel column chromatography (50% ethyl acetate/hexanes) to yield 1.4 g (4.7 mmol) of product **5** as a mixture of isomers. 81% mass recovery. ^{1}H NMR (600 MHz, CHLOROFORM-*D*) δ 7.99 – 7.80 (m, 2H), 7.70 – 7.59 (m, 1H), 7.57 – 7.40 (m, 2H), 5.49 – 5.30 (m, 1H), 4.08 – 3.75 (m, 1H), 3.71 (dt, J = 22.5, 6.4 Hz, 1H), 3.60 – 3.50 (m, 1H), 3.51 – 3.29 (m, 1H), 3.13 – 2.65 (m, 2H), 2.62 – 2.41 (m, 1H), 2.37 – 2.24 (m, 1H), 2.12 – 1.99 (m, 1H), 1.96 – 1.86 (m, 1H), 1.86 – 1.77 (m, 1H), 1.75 – 1.49 (m, 2H), 1.49 – 1.22 (m, 2H). HRMS (EI-TOF): calculated for $C_{17}H_{22}O_3S$ [M+] m/z 307.1368; observed m/z 307.1369.

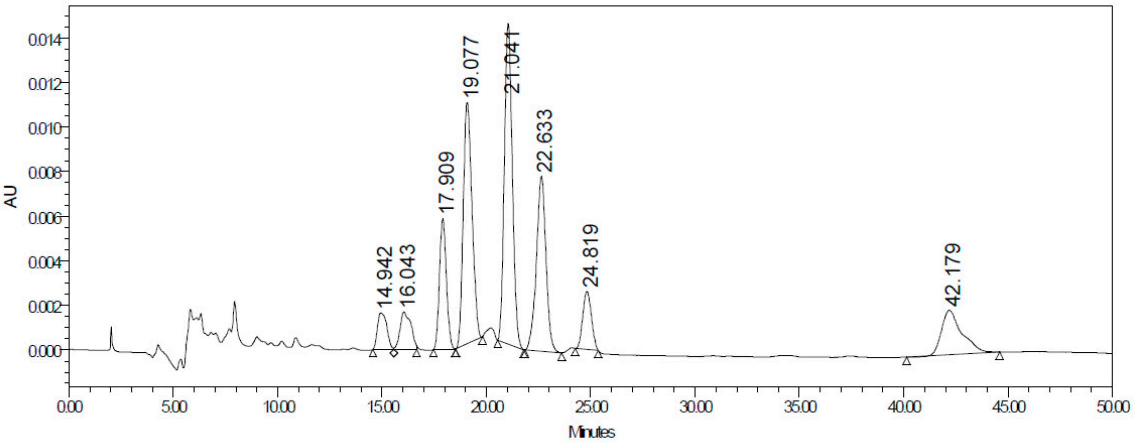

Figure S1: HPLC trace of 5 showing the possible formation of all eight possible pairs of enantiomers.

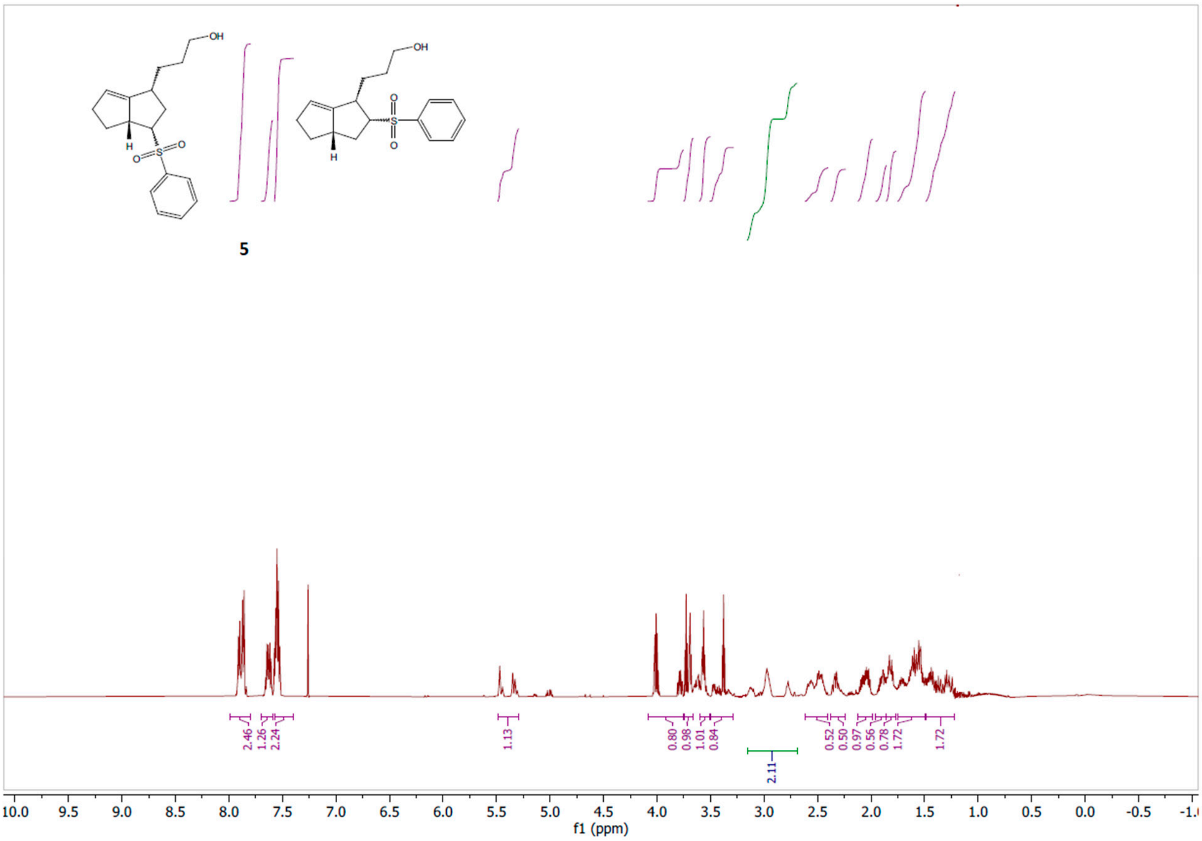

*Figure S2: ^{1}H NMR spectrum of **5** as a mixture of isomers ($CDCl_3$).*

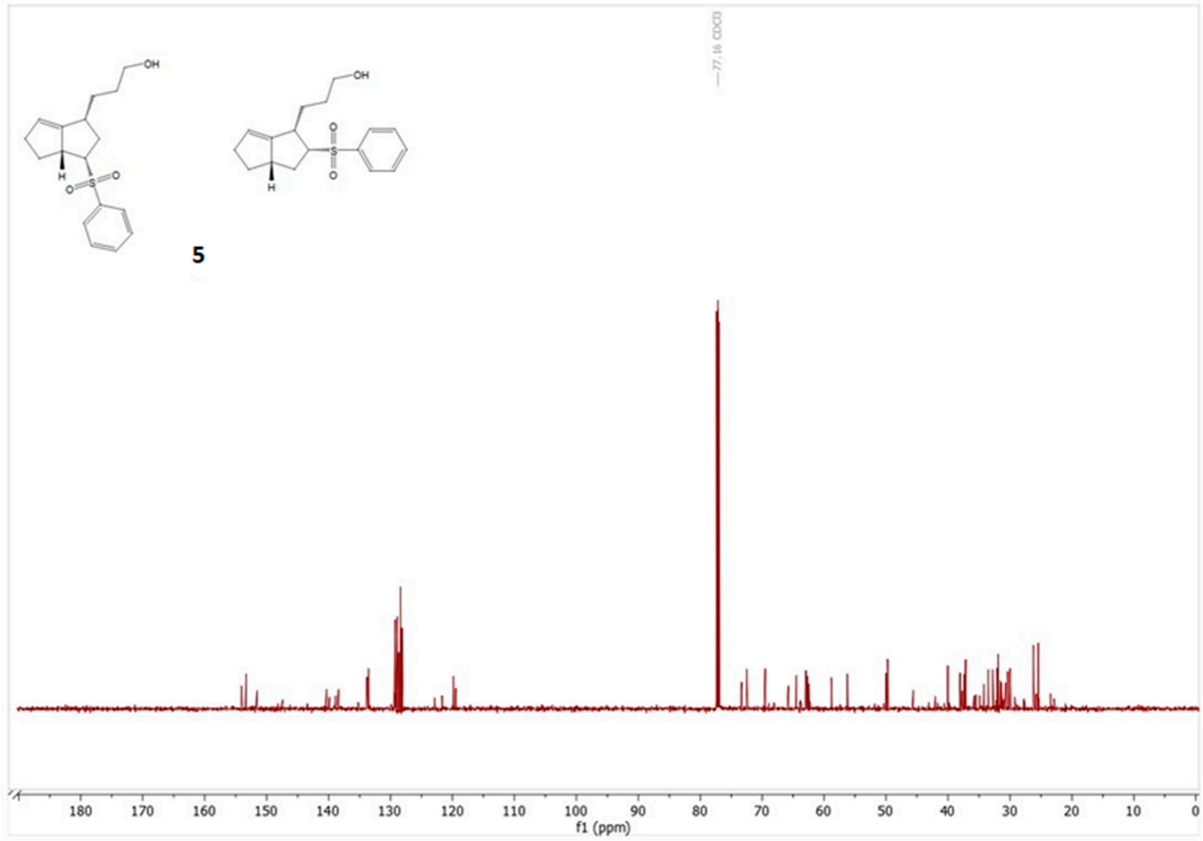

*Figure S3: ^{13}C NMR spectrum of **5** as a mixture of isomers ($CDCl_3$).*

3-[1,2,3,3*a*,4,5-Hexahydropentalen-1-yl]propan-1-ol (**6**):

Crude product **5** (1.1 g 3.56 mmol) was dissolved in 16 mL of methanol in a 100 mL round-bottom flask equipped with a magnetic stir bar. To this was added 2.0 g (14.4 mmol) of disodium phosphate and allowed to dissolve for 10 minutes. An amount of 3.3 g (14.6 mmol) of sodium mercury amalgam 20% was added to the reaction flask all at once. The flask was plugged with a rubber stopper and allowed to stir for 12 hours at rt until completion. Reaction was washed with a saturated aqueous solution of sodium bicarbonate and extracted with 3x20 mL dichloromethane. The organic fractions were combined, dried with Na_2SO_4, and concentrated. The product was purified using silica gel column chromatography (50% ethyl acetate/hexanes) to yield 0.44 g (2.65 mmol, 75% yield) of reduced trap product **6** at a ratio of 5:1 of the two diastereomers determined by ^1H NMR integration of the vinyl proton.

Isomer 1 (major): ^1H NMR (600 MHz, $CDCl_3$ δ 5.20 (br s, 1H; H2), 3.54 (t, J = 4.0 Hz, 2H; H11α and β), 3.16 (br m, 1H; H12), 2.90 – 2.75 (m, 1H; H5), 2.60 – 2.46 (m, 1H; H8), 2.45 – 2.30 (m, 2H; H3α or β and H9α or β), 2.30 – 2.18 (m, 1H; H3α or β), 2.08 – 1.95 (m, 2H; H6α or β and H9α or β), 1.79 (td, J = 12.1, 8.0, 5.1 Hz, 1H; H4α or β), 1.63 – 1.40 (m, 3H; H4α or β, H7α or β, and H10α or β), 1.37 – 1.15 (m, 2H; H6α or β and H7α or β), 1.09 – 0.90 (m, 1H;

H10α or β). ^{13}C NMR (151 MHz, $CDCl_3$) δ 158.62 (C1), 116.37 (C2), 62.74 (C11), 51.62 (C5), 37.67 (C8), 37.15 (C7), 36.45 (C6), 34.75 (C3), 32.84 (C10), 30.44 (C4), 29.88 (C9).

Isomer 2 (minor): ^{1}H NMR (600 MHz, $CDCl_3$) δ 4.92 (br s, 1H; H2), 3.54 (t, J = 4.0 Hz, 2H; H11α and β), 3.16 (s, 1H; H12), 2.90 – 2.75 (m, 1H; H5), 2.60 – 2.46 (m, 1H; H8), 2.45 – 2.30 (m, 2H; H3α or β and H9α or β), 2.30 – 2.18 (m, 1H; H3α or β), 2.08 – 1.95 (m, 2H; H6α or β and H9α or β), 1.79 (td, J = 12.1, 8.0, 5.1 Hz, 1H; H4α or β), 1.63 – 1.40 (m, 3H; H4α or β, H7α or β, and H10α or β), 1.37 – 1.15 (m, 2H; H6α or β and H7α or β), 1.09 – 0.90 (m, 1H; H10α or β). ^{13}C NMR (151 MHz, $CDCl_3$ δ 157.63 (C4), 118.24 (C2) 62.58 (C11), 51.20 (C5), 39.74 (C6) 36.64 (C7), 36.59 (C8) 32.28 (C1), 32.04 (C10), 31.33 (C3), 31.16 (C9). HRMS (EI-TOF): calculated for $C_{11}H_{18}O$ [M+] m/z 166.13577; observed m/z 166.13520.

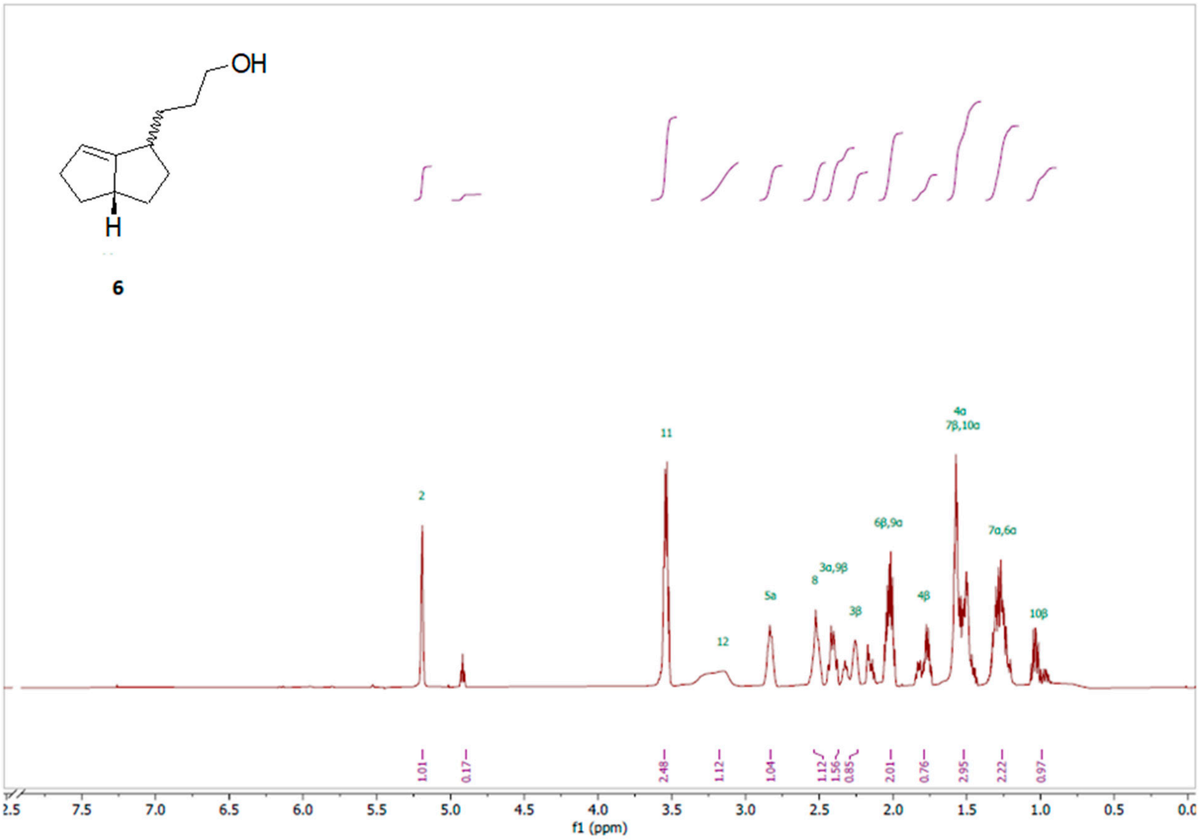

*Figure S4: ^{1}H NMR spectrum of **6**, two isomers ($CDCl_3$).*

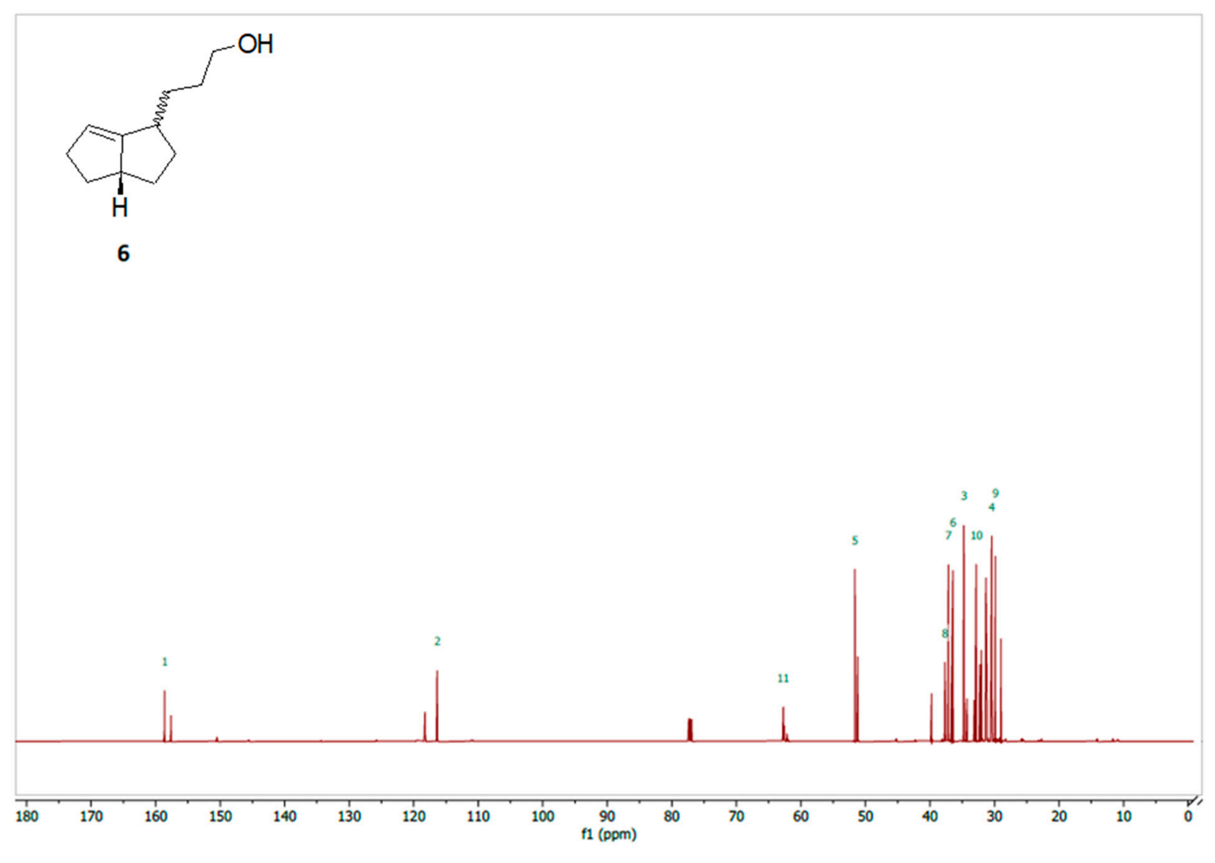

*Figure S5: ^{13}C NMR spectrum of **6**, two isomers ($CDCl_3$).*

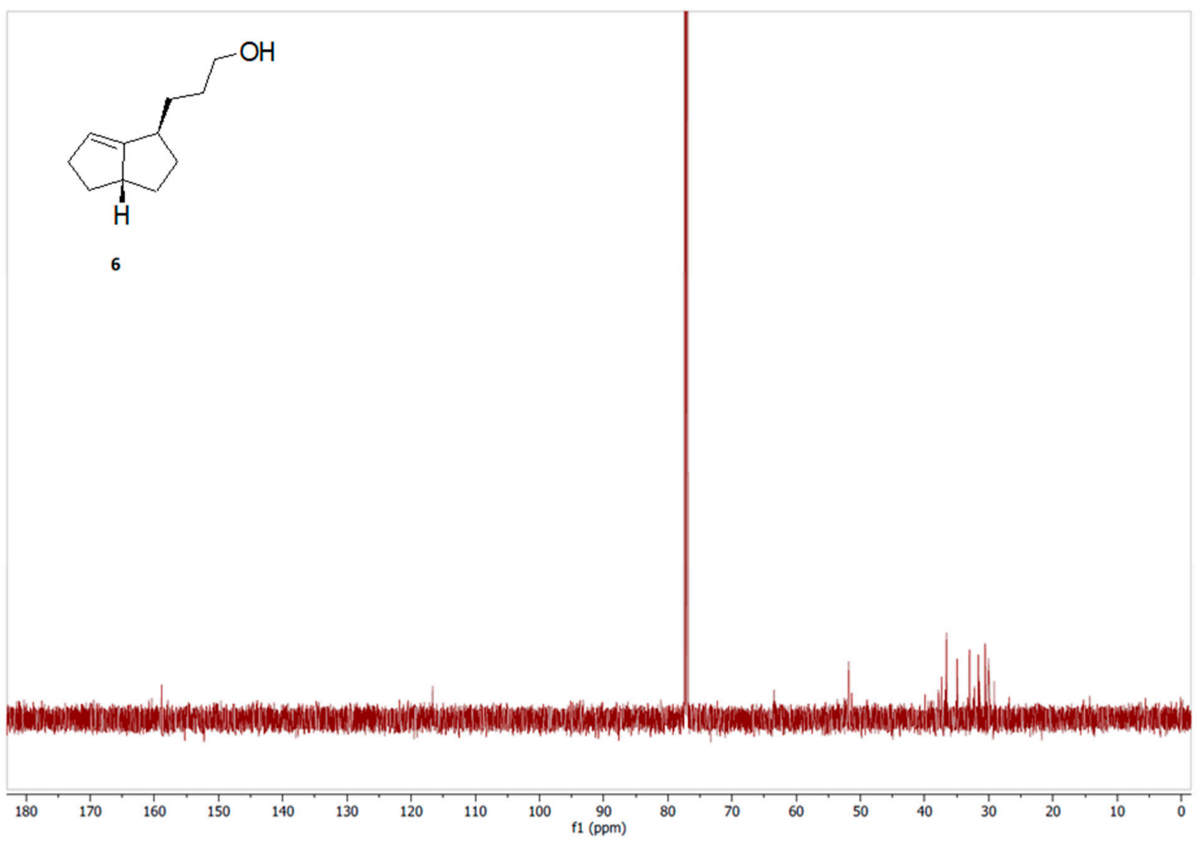

*Figure S6: ^{13}C NMR spectrum of **6** (syn-isomer) ($CDCl_3$).*

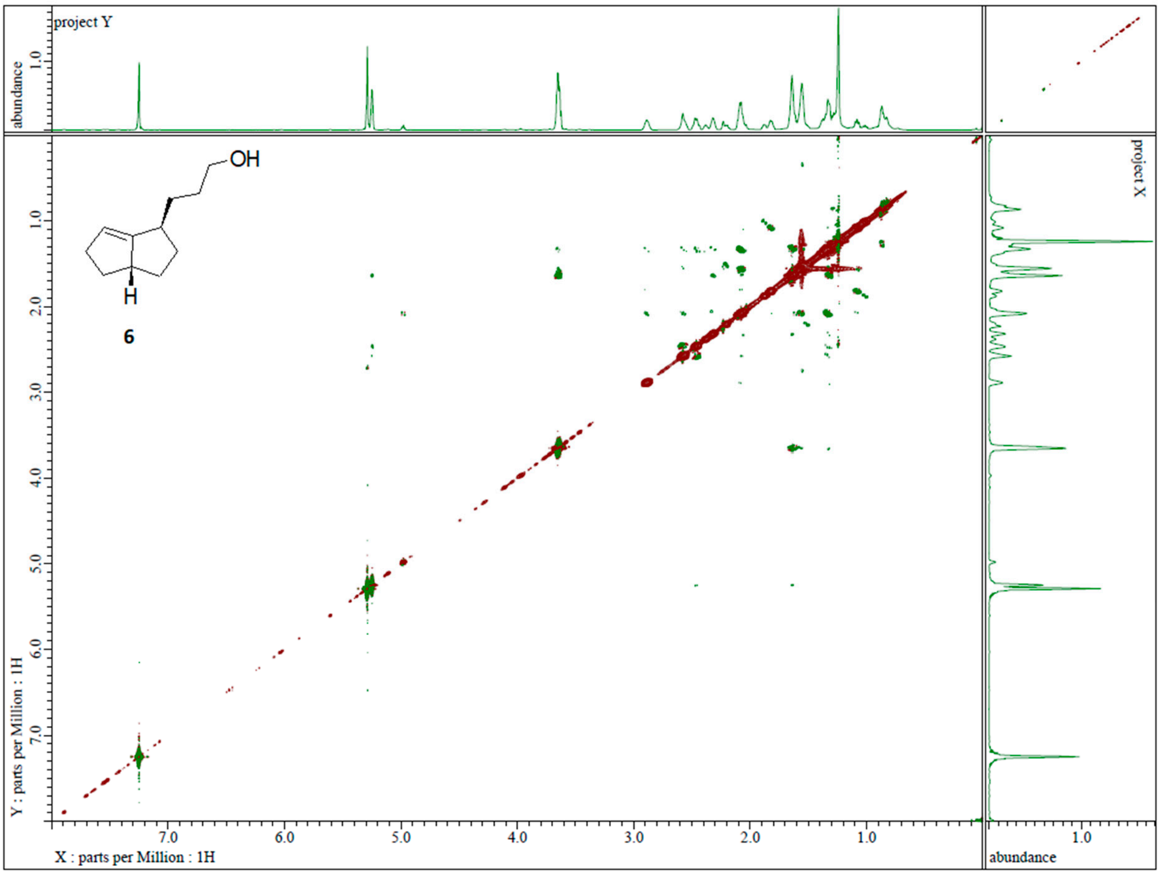

*Figure S7: NOESY spectrum of compound **6** ($CDCl_3$).*

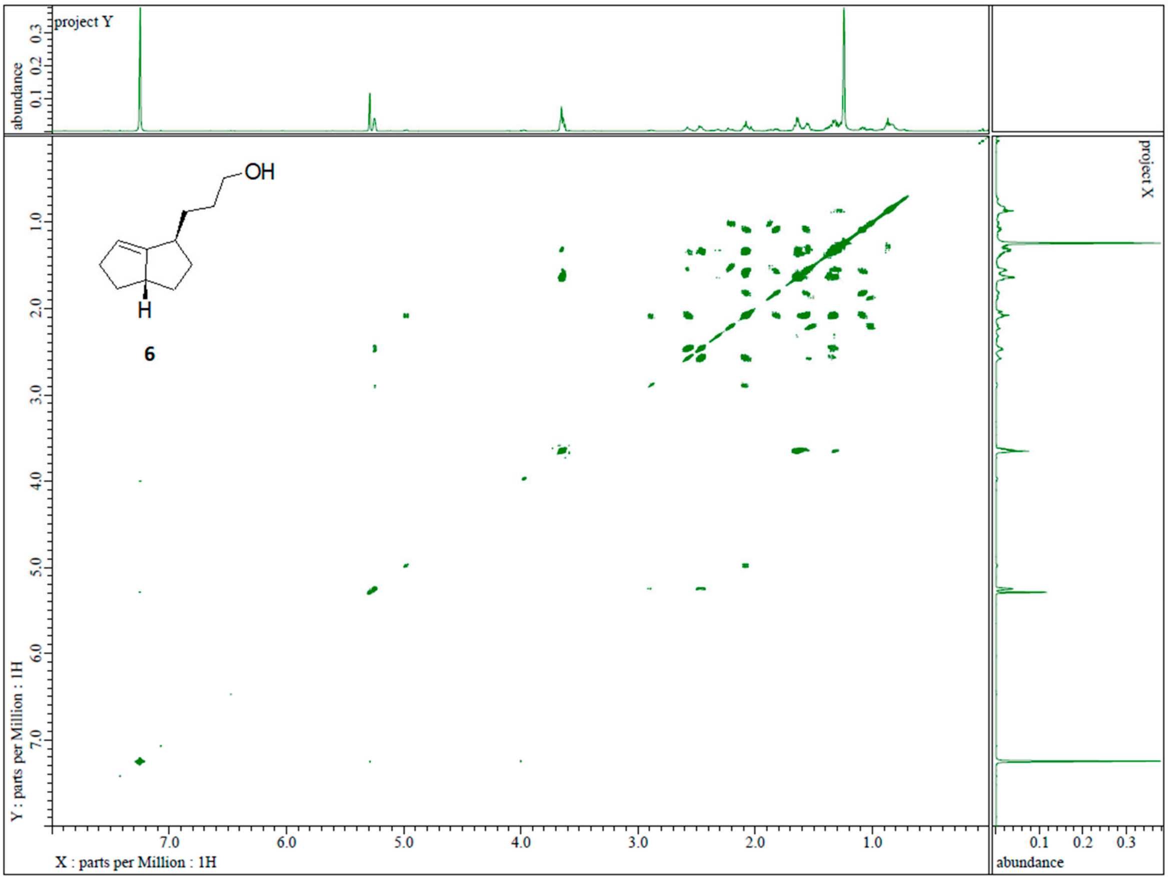

*Figure S8: COSY spectrum of compound **6** ($CDCl_3$)..*

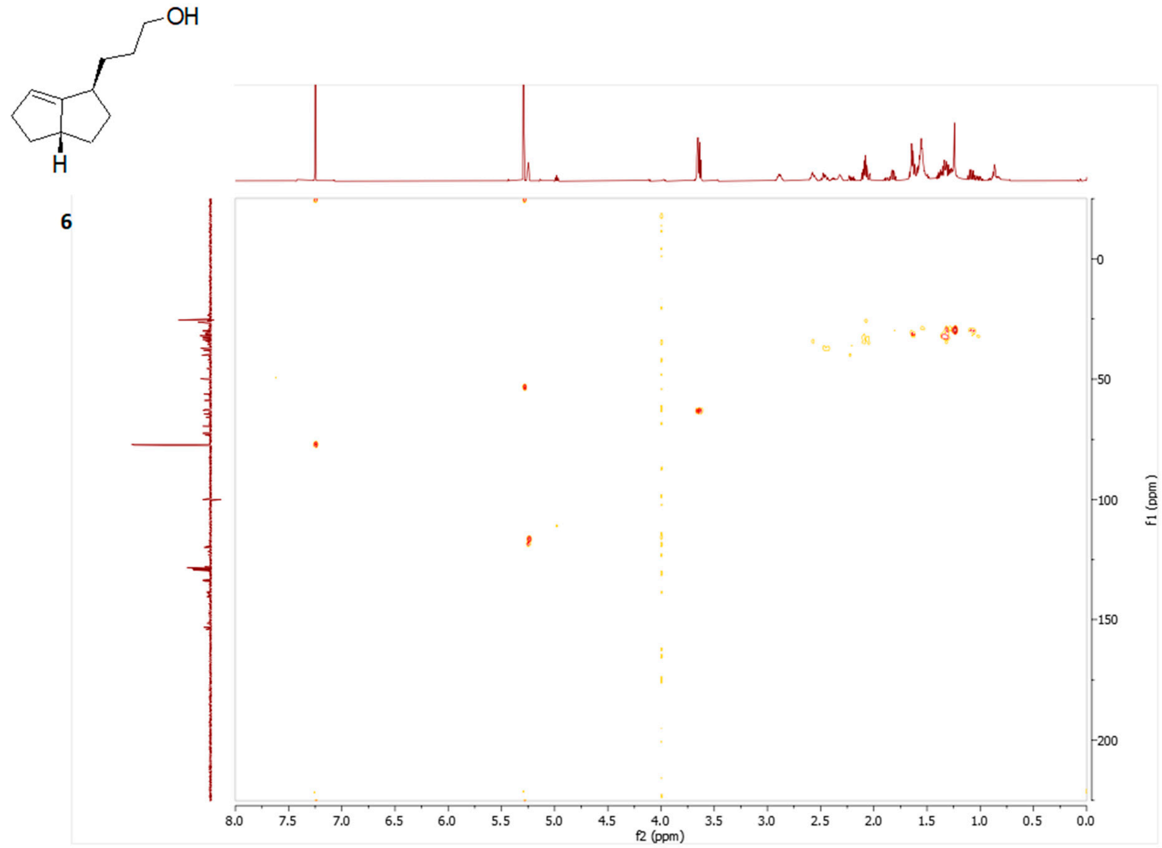

*Figure S9: HMQC spectrum of compound **6** ($CDCl_3$).*

3-[Hexahydro-1*aH*-pentaleno[1,6*a*-*b*]oxiren-6-yl]propan-1-ol (**7**):

Compound **6** (0.44 g, 2.64 mmol) was dissolved in 20 mL of dichloromethane in a 100 mL round-bottom flask and cooled in an ice bath and stirring after adding 0.56 g (6.39 mmol) of sodium carbonate. The reaction was allowed to cool to 0 °C for 20 minutes. mCPBA (0.54 g, 3.11 mmol) in 10 mL of dichloromethane and was added dropwise into the reaction mixture over a period of 10 minutes. Reaction continued for 15 more minutes while cooled and was complete according to TLC analysis. Saturated sodium bicarbonate (20 mL) was added, and the reaction mixture was extracted with 3x20 mL of diethyl ether. The combined organic layers were dried with Na_2SO_4 and concentrated leaving 0.35 g (1.93 mmol, 73% yield) of epoxide **7**. The ratio of the cis to trans isomer was 5:1 respectively confirmed by ^{1}H NMR integration of C**H**-O. ^{1}H NMR (600 MHz, $CDCl_3$) δ 3.46 (br t, 2H), 3.30 (major isomer) and 3.12 (minor isomer) (s, 5:1 ratio, 1H, CH-O), 3.20 (bs, 1H, OH), 2.18 – 2.08 (m, 2H), 1.99 – 1.92 (m, 1H), 1.86 – 1.79 (m, 1H), 1.72 – 1.61 (m, 2H), 1.55 – 1.42 (m, 3H), 1.41 – 1.33 (m, 2H), 1.30 – 1.23 (m, 1H), 1.17 – 1.08 (m, 1H), 1.05 – 1.00 (m, 1H). ^{13}C NMR (151 MHz, $CDCl_3$ δ Isomer 1: 80.93, 62.28, 61.11, 41.76, 34.73, 31.03, 28.94, 28.21, 26.72, 26.54, 26.23; Isomer 2: 79.75, 65.12, 57.39, 40.55,

35.51, 30.35, 29.46, 27.29, 26.44, 26.36, 25.76. HRMS (EI-TOF): calculated for $C_{11}H_{18}O_2$ [M+] m/z 182.1307; observed m/z 182.1302.

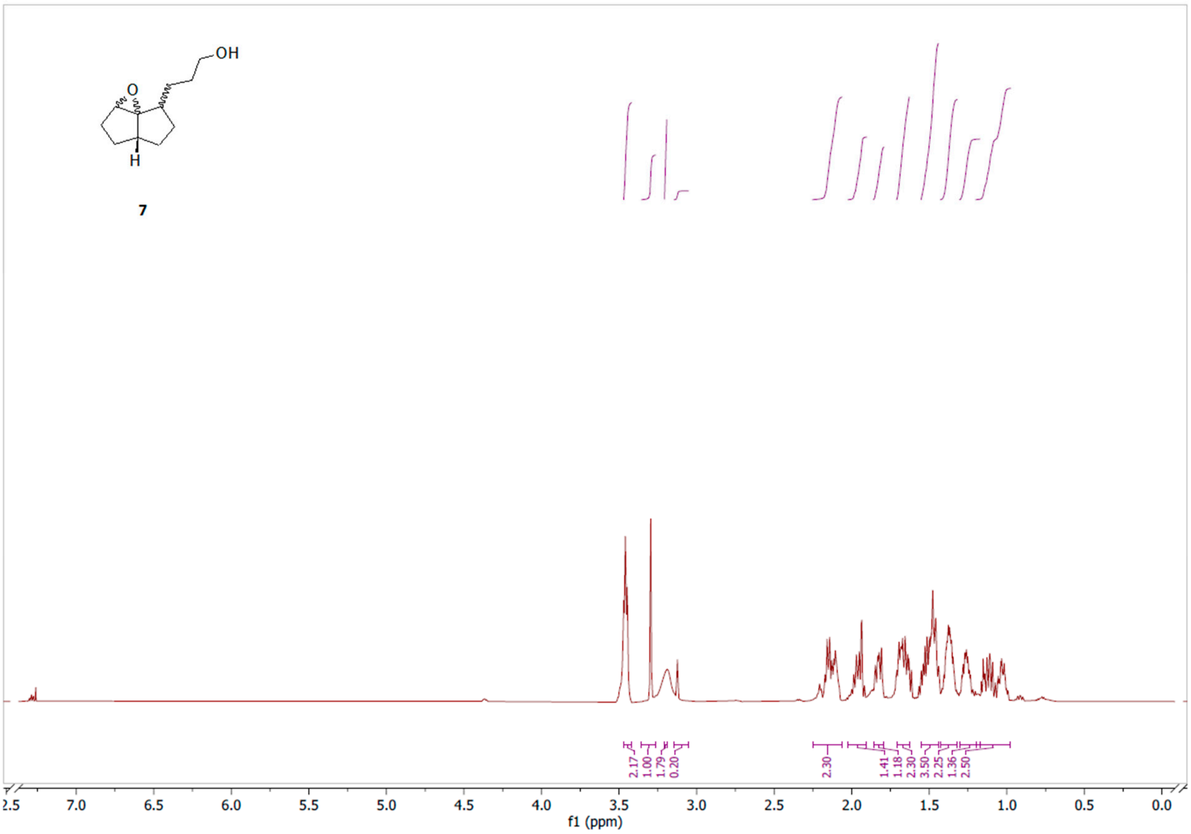

*FigureS10: ^{1}H NMR spectrum of **7**, two isomers ($CDCl_3$).*

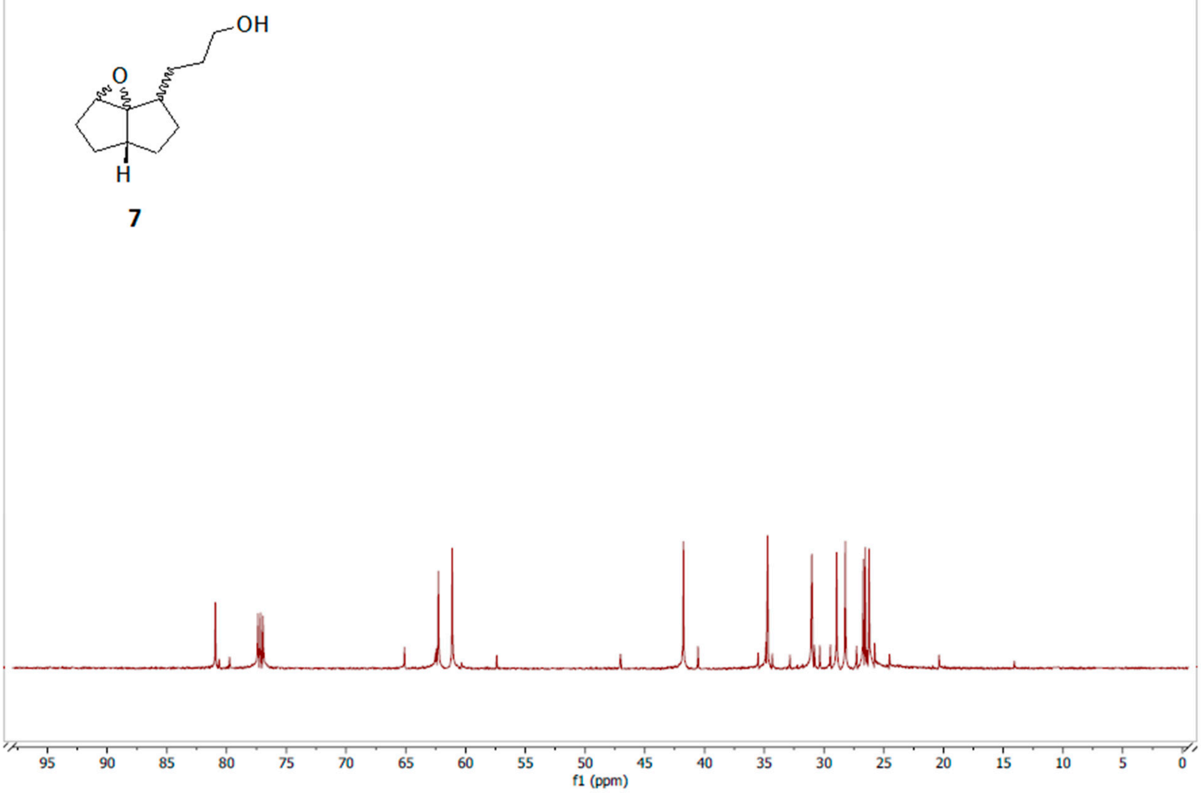

*Figure S11: ¹³C NMR spectrum of **7**, two isomers (CDCl₃).*

tert-butyl{3-[Hexahydro-1a*H*-pentaleno[1,6*a*-*b*]oxiren-6-yl]propoxy}dimethylsilane (7-TBDMS ether):

Epoxide **7** (0.19 g, 1.05mmol) was dissolved in dry DMF under nitrogen atmosphere at rt. Imidazole (2.5 mmol) and TBDMSCl (1.3 mmol) were added and the reaction stirred for 18 hours. The reaction was diluted with saturated brine and extracted with hexanes (3x20 mL). Combined organic layer was dried over Na_2SO_4, filtered and concentrated. Column chromatography on silica gel (5% EtOAc/hexanes) yielded 0.24 g (0.81 mmol, 77% yield) of protected epoxide. ^{1}H NMR (600 MHz, $CDCl_3$) δ 3.61 – 3.53 (m, 2H), 3.36 (major isomer) and 3.20 (minor isomer) (d, 2.0 Hz, 1H), 2.25 (dtd, J = 10.8, 8.4, 2.4 Hz, 1H), 2.19 (td, J = 9.7, 4.8 Hz, 1H), 2.05 (ddq, J = 13.2, 10.1, 8.4 Hz, 1H), 1.94 – 1.90 (m, 1H), 1.81 – 1.69 (m, 2H), 1.68 – 1.62 (m, 1H), 1.60 – 1.50 (m, 2H), 1.49 – 1.38 (m, 2H), 1.37 – 1.30 (m, 1H), 1.26 – 1.17 (m, 1H), 1.13 – 1.04 (m, 1H), 0.88 (s, 9H), 0.03 (s, 6H). ^{13}C NMR (151 MHz, $CDCl_3$) δ Isomer 1: 80.99, 63.46, 61.13, 42.06, 35.12, 31.56, 29.26, 28.63, 27.05, 26.83, 26.58, 26.13, 18.5, -5.12. MS (ESI): calculated for $C_{17}H_{32}O_2Si$ [M+] m/z 296.5202; found [M+Na] m/z 319.5.

3-(3-{[*tert*-butyl(dimethyl)silyl]oxy}propyl)-2,3,6,6a-tetrahydropentalen-3a(1*H*)-ol (**8**):

To a 50 mL flame-dried, round bottom-flask with 3 mL of 2-butanol, under nitrogen gas was added 0.15 mg (0.46 mmol) of diphenyl diselenide and it was allowed about 10 minutes to dissolve before adding 35.2 mg (0.93 mmol) of sodium borohydride in small increments over about 20 minutes and a color change from yellow to clear, and bubbling occurred in the flask as the sodium borohydride was being added in. Once the color change was complete, protected epoxide was injected into the rubber stopper on top of the flask in 1 mL of 2-butanol and the flask was quickly transferred to a reflux condenser to be refluxed at 100 °C for 22 hours under nitrogen atmosphere. After completion according to tlc, the reaction flask was removed from reflux, cooled over ice and then 2 mL of tetrahydrofuran was added. After cooling to 0 °C, 0.87 mL of 35% H_2O_2 was added to the reaction dropwise over 45 minutes. After bubbling stopped, the reaction was placed on an oil bath and refluxed at 100 °C for 20 hours at which point the elimination was complete according to TLC analysis. To the reaction flask was added 20 mL H_2O and extraction was done with 3x20 mL of diethyl ether. The organic layers were washed with sodium carbonate solution and the resulting organic layers were dried with Na_2SO_4, filtered, and concentrated down to give 0.15 g (0.52 mmol, 64% yield) of allylic alcohol **8**. ^{1}H NMR (600 MHz, $CDCl_3$) δ 5.86 (major isomer) and 5.68 (minor isomer) (m, 1H) 5.55 (m, 1H), 3.66 – 3.57 (m, 2H; H11), 3.35 (s, 1H; -OH), 2.79 (major isomer) and 2.70 (minor isomer) (qt, J = 17.6, 8.9, 2.3 Hz, 1H; H5), 2.38 (ddd, J = 10.9, 6.2, 2.4 Hz, 1H; H8), 2.36 – 2.24 (m, 1H), 2.00 – 1.83 (m, 3H), 1.78 – 1.73 (m, 1H), 1.66 – 1.50 (m, 2H), 1.41 – 1.34 (m, 1H), 1.22 – 1.15 (m, 1H), 1.06 – 0.96 (m, 1H), 0.88 (s, 9H), 0.06 (s, 6H). ^{13}C NMR (151 MHz, $CDCl_3$) δ Isomer 1: 135.33 (C2), 132.06 (C3), 63.83 (C1), 50.85 (C11), 49.14 (C5), 41.32 (C8), 32.09 (C4), 31.40 (C9), 29.78 (C10), 29.48 (C6), 27.00 (C7), 26.11 (C16, C15, C17), 18.49 (C14), -5.14 (C12, C13); Isomer 2: 135.78 (C2), 131.88 (C3), 63.67 (C1), 51.83 (C11), 49.57 (C5), 42.06 (C8), 32.92 (C4), 32.02 (C9), 29.82 (C10), 29.03 (C6), 25.23 (C7), 25.78 (C15, C16, C17), 18.09 (C14), -3.46 (C12, C13). HRMS (EI-TOF): calculated for $C_{17}H_{32}O_2Si$ [M+] m/z 296.2172; observed m/z 296.2179.

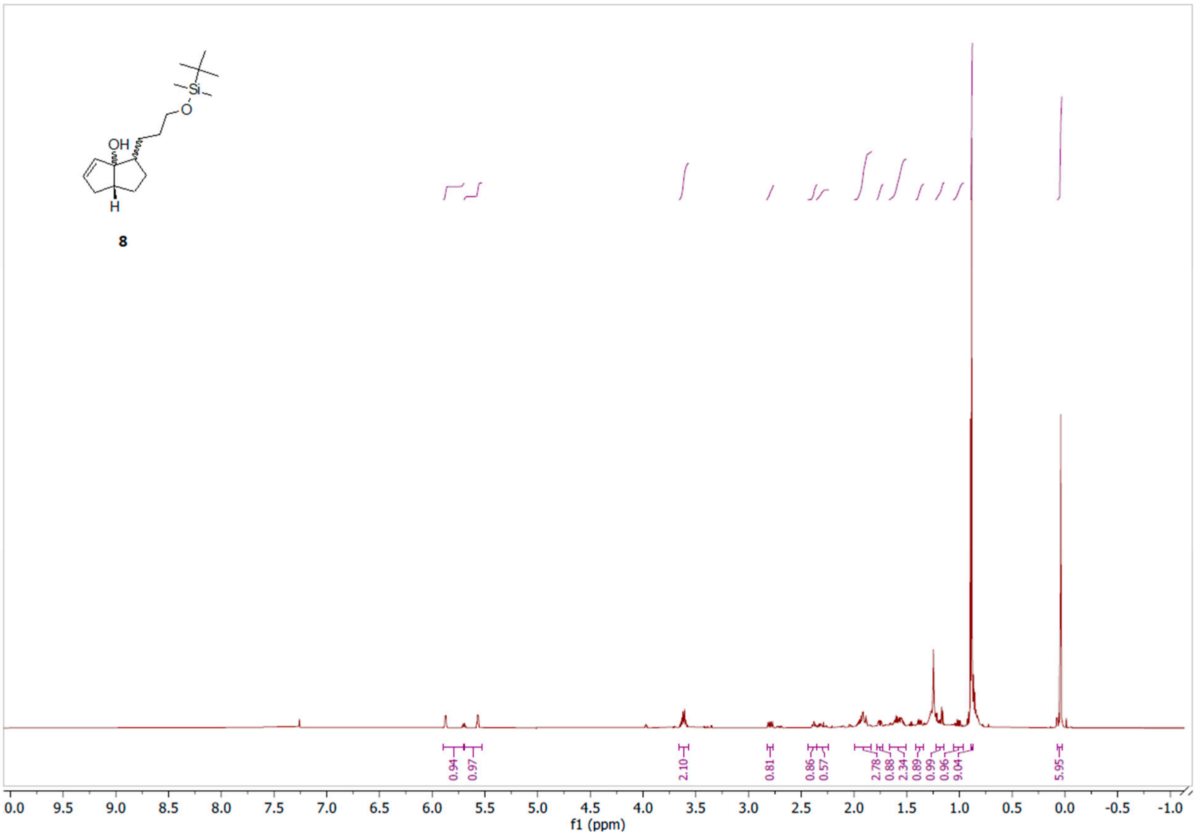

*Figure S12: ^{1}H NMR spectrum of **8**, two isomers ($CDCl_3$).*

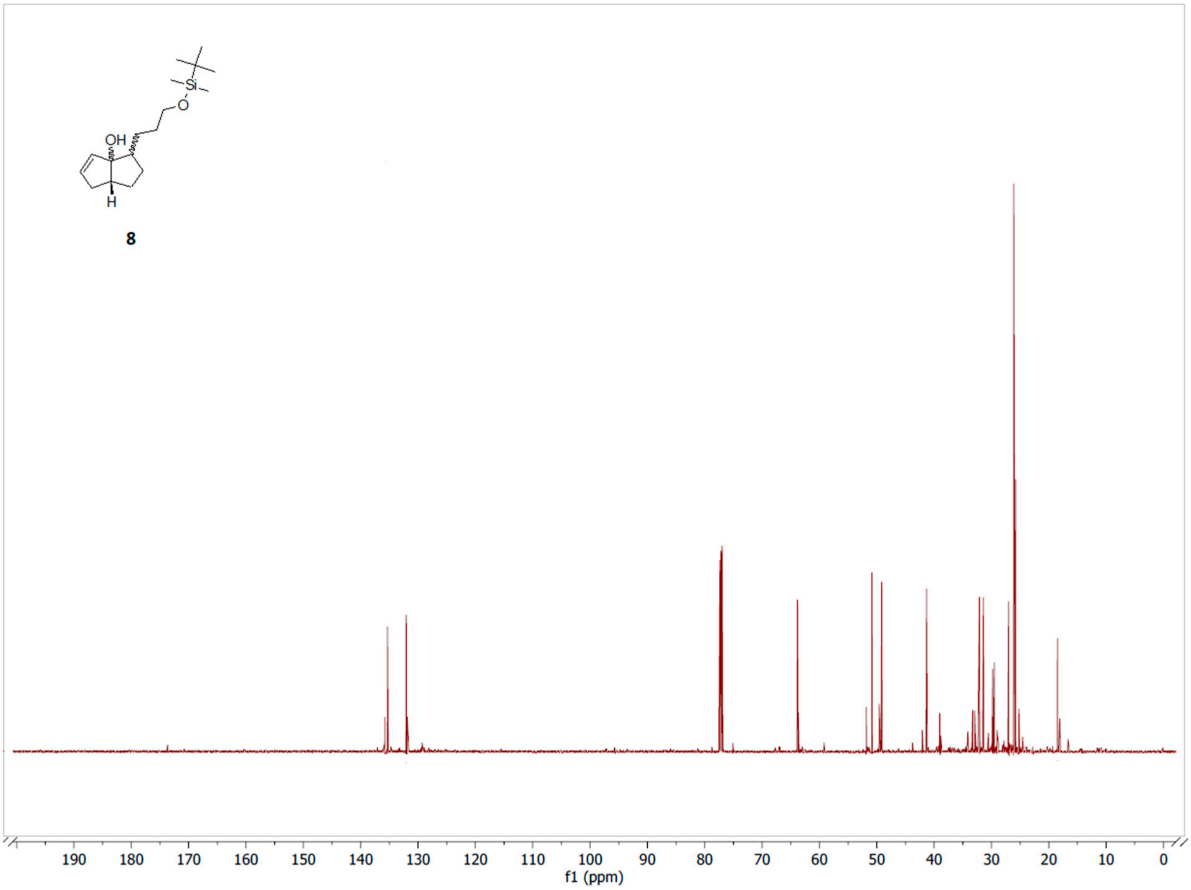

*Figure S13: ^{13}C NMR spectrum of **8**, two isomers ($CDCl_3$).*

6-(3-{[*tert*-butyl(dimethyl)silyl]oxy}propyl)-3a,4,5,6-tetrahydropentalen-2(3*H*)-one (**9**):

In 25 mL round-bottom flask equipped with a stir bar and under nitrogen atmosphere was added 3 mL of dry dichloromethane at rt. Celite (0.5 g) and PCC (230 mg) were suspended in the dichloromethane and stirring was begun. Allylic alcohol **8** (0.15 g, 0.52 mmol) was dissolved in 1 mL of dichloromethane was injected into the reaction flask via syringe. The reaction turned black and tarry almost immediately upon addition of the allylic alcohol. After 6 hours the reaction was complete according to TLC analysis at which point it was decanted with ether and the flask was triturated and decanted with ether several times. This solution was run directly through a plug of silica gel on a glass fritted filter to remove any solids. The filtered organics were concentrated with a rotary evaporator and chromatographed via flash column chromatography (20% ethyl acetate/hexanes) to yield 0.6 g (0.20 mmol, 40% yield) of the of α, β- unsaturated ketone **9**. ^{1}H NMR (600 MHz, $CDCl_3$) δ 5.86 (t, J = 2.1 Hz, 1H; H2), 3.63 (t, J = 6.3 Hz, 2H; H11), 2.95 (dddt, J = 11.3, 8.4, 5.7, 2.7 Hz, 1H; H5), 2.80 (tdd, J = 15.7, 12.5, 6.2 Hz, 1H; H8), 2.59 (dd, J = 6.4, 6.4 Hz, 1H), 2.22 (dtd, J = 13.2, 10.3, 7.7 Hz, 1H), 2.16 – 2.10 (m, 1H), 2.06 (d, J = 0.0 Hz, 1H), 1.79 – 1.67 (m, 2H), 1.67 – 1.53 (m, 2H), 1.38 (dtd, J = 13.3,

9.3, 5.7 Hz, 1H), 1.23 – 1.14 (m, 1H), 0.88 (s, 9H), 0.04 (s, 6H). ^{13}C NMR (151 MHz, $CDCl_3$) δ 195.42, 123.52, 123.47, 63.03, 46.26, 42.77, 38.70, 31.92, 31.24, 30.04, 29.68, 26.07, 18.46, -5.17. HRMS (EI-TOF): calculated for $C_{17}H_{30}O_2Si$ [M+] m/z 294.2015; observed m/z 294.2023. UV Spec λmax=203 (0.9814).

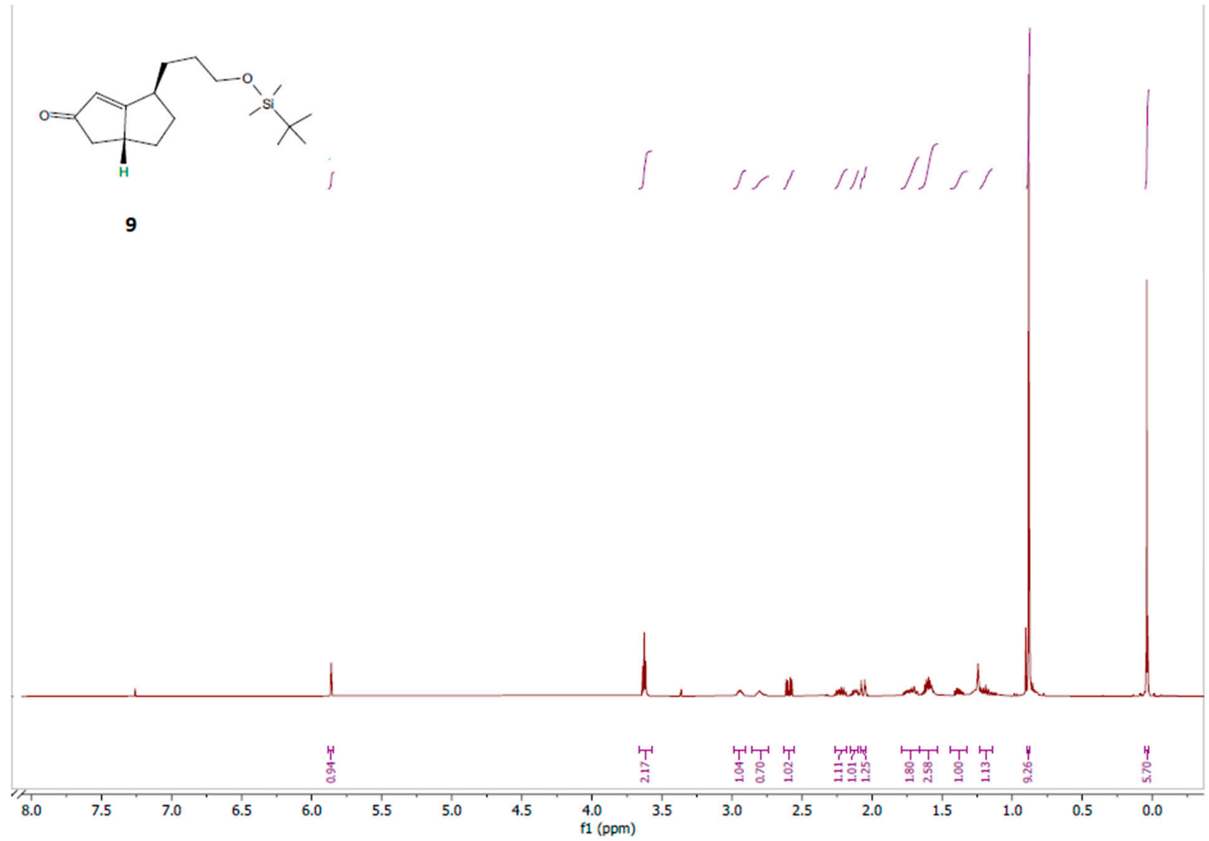

*Figure S14: ^{1}H NMR spectrum of **9** ($CDCl_3$).*

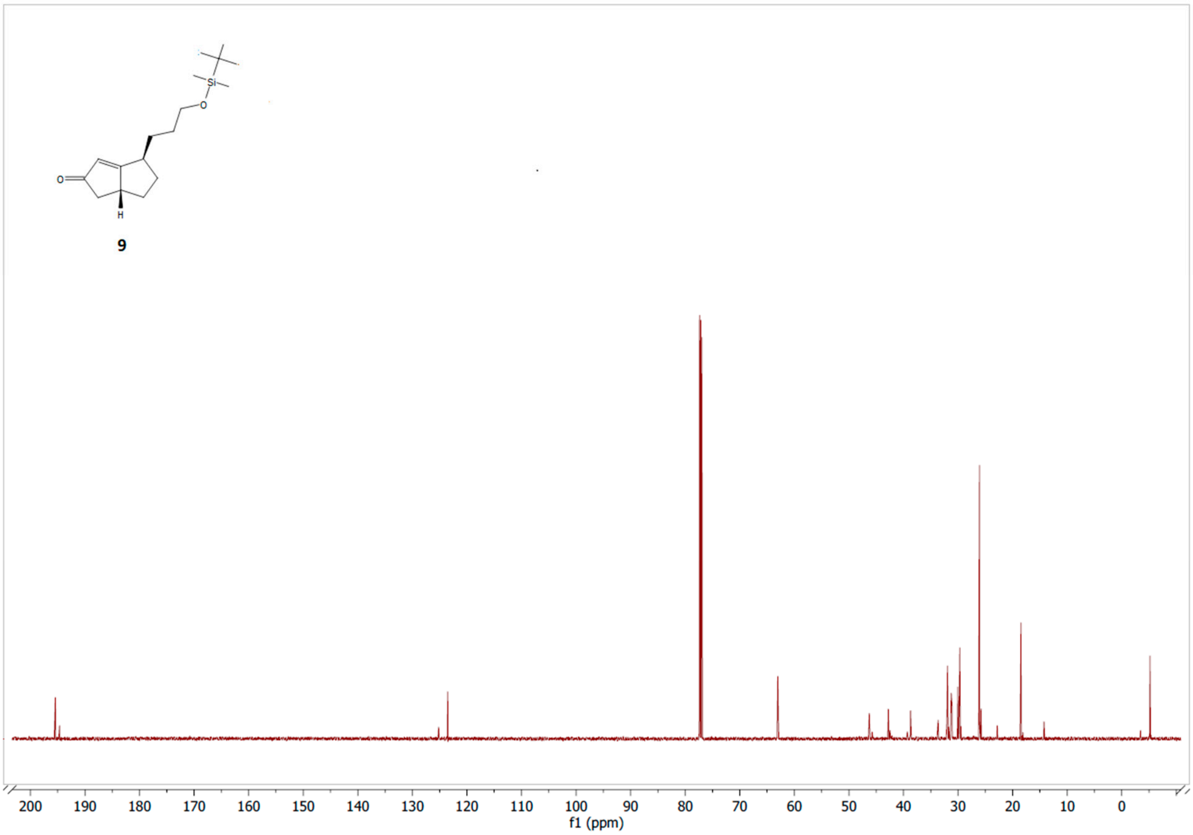

Figure S15:[13]*C NMR spectrum of* ***9*** *(CDCl*$_3$*).*

8-(3-{[*tert*-butyl(dimethyl)silyl]oxy}propyl)-2-methylideneoctahydrocyclopenta[*c*]pentalen-4(5*H*)-one (**10**):

In a 10 mL flame-dried, round-bottom flask equipped with a stir bar was added 1.5 mL of dry tetrahydrofuran, which was thoroughly degassed under nitrogen atmosphere. To the degassed THF was added 15.7 mg (0.0136 mmol) of tetrakis(triphenylphosphine)palladium(0) followed by 2.0 mg (0.0051 mmol) of 1,2-Bis(diphenylphosphino)ethane (dppe) and 21.05 mg (0.113 mmol) of 2-(trimethylsilylmethyl)allyl acetate. After 5 minutes of stirring, 33 mg (0.17 mmol) of the α, β- unsaturated ketone **9** dissolved in 0.5 mL of degassed, dry THF was injected into the reaction mixture with a syringe under a nitrogen atmosphere. The reaction was then refluxed at 75 °C for 20 hours at which point it was complete according to TLC analysis. The reaction was diluted with 10 mL of dichloromethane, washed with 10 mL of saturated brine, and extracted with 2x10 mL dichloromethane. The organic layers were combined, dried with Na_2SO_4, concentrated in a rotary evaporator, and then chromatographed with flash chromatography on silica gel (10% ethyl acetate/hexanes) to yield 12 mg (0.035 mmol, 31% yield) of angular triquinane **10**. ^{1}H NMR (600 MHz, $CDCl_3$ δ ^{1}H NMR (600 MHz, $CDCl_3$) δ ([4.73 and 4.56 (br s) C12 H minor isomer], [4.72 and 4.59 (br s) C12 H major isomer] 2H), 3.97 (m, 1H), 3.61 (t, J = 6.3 Hz, 2H), 3.06 (d, J

= 16.1 Hz, 1H), 2.92 – 2.77 (m, 2H), 2.64 (td, J = 18.1, 6.2 Hz, 2H), 2.32 (q, J = 5.8 Hz, 1H), 2.29 – 2.24 (m, 1H), 1.76 – 1.65 (m, 7H), 1.35 (td, J = 6.8, 1.9 Hz, 2H), 0.90 (s, 9H), 0.04 (s, 6H). MS (ESI): M+ calculated for $C_{21}H_{36}O_2Si$ [M+] m/z 348.247908; found [M+H] m/z 349.1; [M+Na] m/z 371.1.

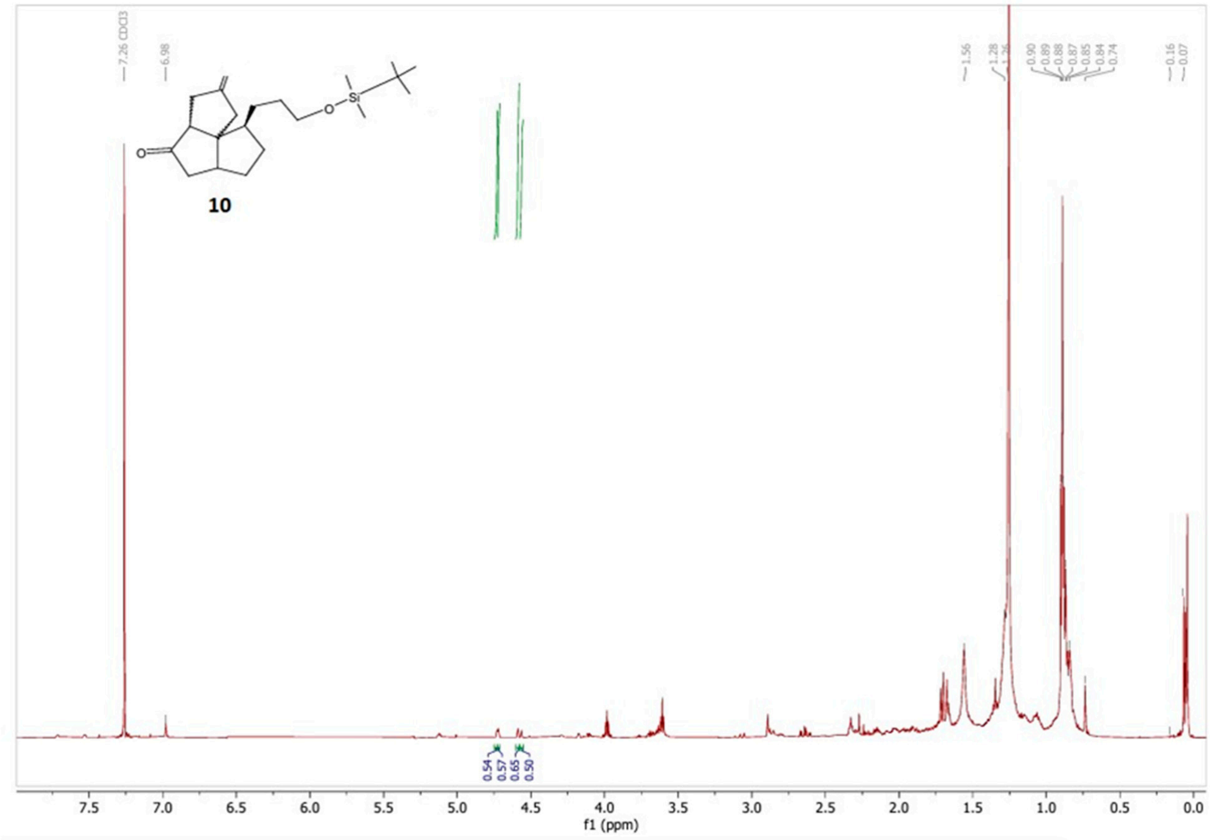

*Figure S16: ^{1}H NMR spectrum of **10** mixture of at least two isomers($CDCl_3$).*

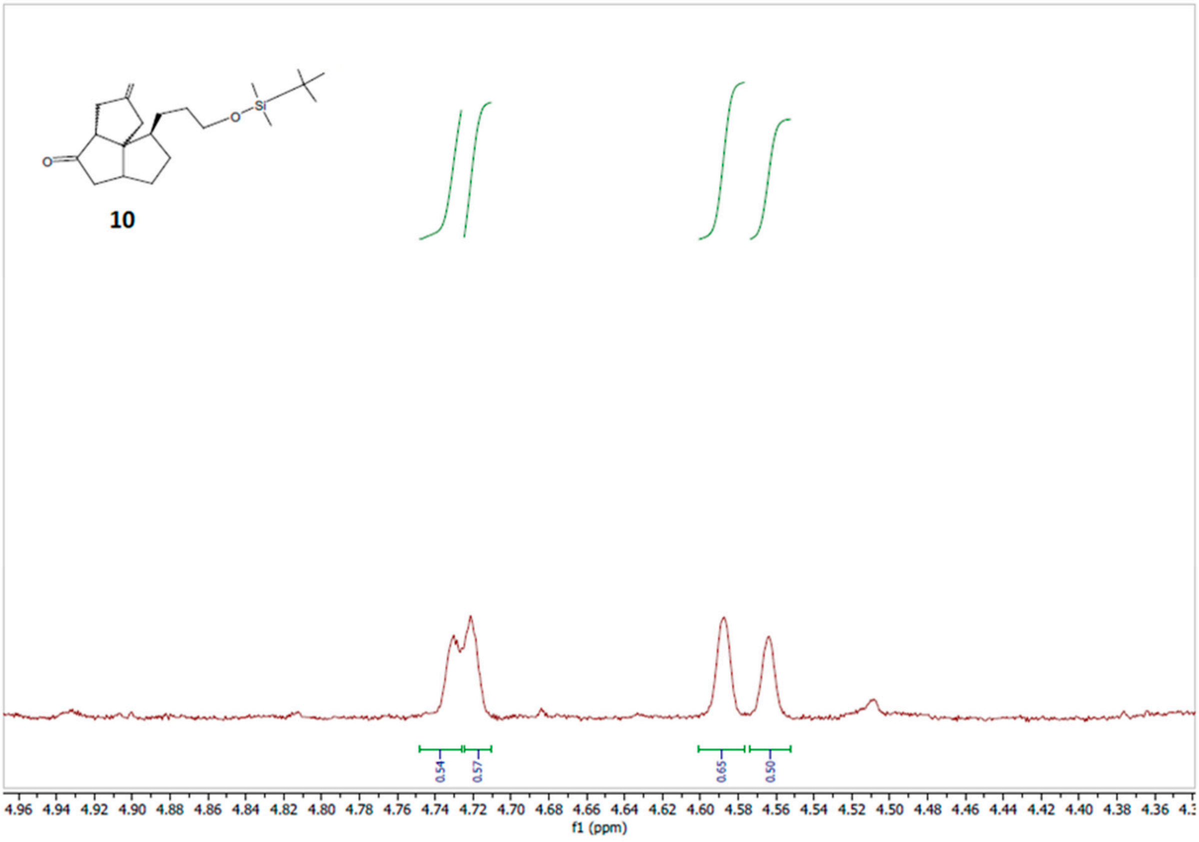

*Figure S17: ^{1}H NMR vinyl region blow up of angular triquinane **10** showing two pairs of methylene vinyl resonances.*

5,5-dimethyl-6',6'a-dihydro-1'*H*-spiro[[1,3]dioxane-2,2'-pentalen]-5'(3'*H*)-one **(11):**

In a 100 mL flame-dried, round-bottom flask was added 28 mL of dry dichloromethane. 0.48 g (2 mmol) of 5,5-dimethyltetrahydro-1'H-spiro[[1,3]dioxane-2,2'-pentalen]-5'(3'H)-one was suspended in the dichloromethane and cooled to -78 °C for 30 min under nitrogen atmosphere. 0.84 mL (6 mmol) of triethylamine was injected into the reaction dropwise. 0.57 mL (4 mmol) of Iodotrimethylsilane was then injected into the reaction. The reaction was allowed to continue for 30 min at -78 °C. After 30 min, 2.8 mL of saturated sodium bicarbonate was added to the reaction and the reaction was allowed to warm to room temperature over a period of 30 min with rigorous stirring. The organic layer was then separated from the aqueous layer and the aqueous layer extracted with 3x10 mL diethyl ether. Organic layers were combined and dried with magnesium sulfate, filtered, and concentrated. 442 mg of palladium acetate was dissolved into 22 mL of dry acetonitrile in a 100 mL round bottom flask. Crude enol silyl ether was then dissolved in 3 mL of dry acetonitrile and injected into the palladium acetate solution under nitrogen gas. The reaction was allowed to stir for 2 hours at room temperature. Upon completion, the reaction mixture was filtered through a plug of fluorosil and eluted with 50 mL of diethyl ether. The eluent was concentrated and then silica gel column chromatography was performed in 1:9 ethylacetate:petroleum ether to yield 370.3 mg of enone ketal (**11**). 78% yield. M+ calculated for

$C_{13}H_{18}O_3$ 222.28022; found M+H=223.3, M+Na=245.3. ^{1}H NMR spectrally consistent with data in literature [13].

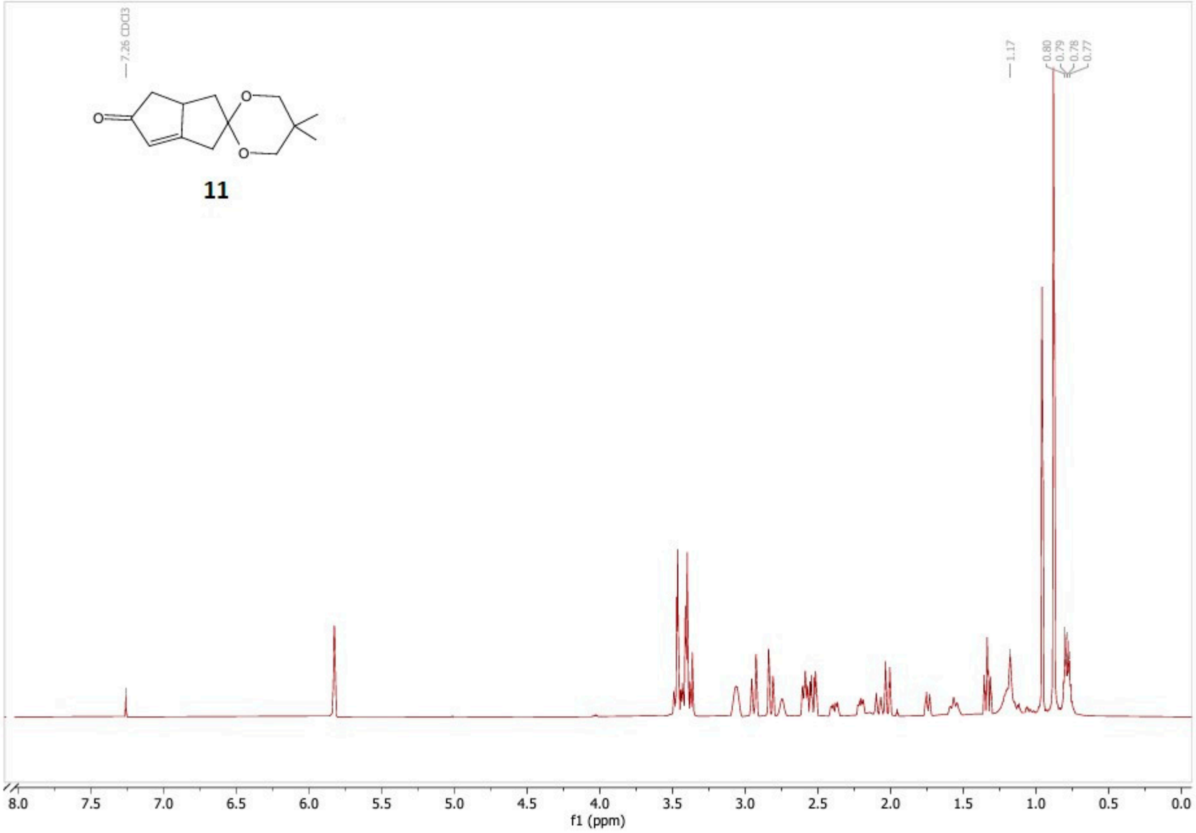

*Figure S18: ^{1}H NMR spectrum of **11** ($CDCl_3$).*

5',5'-dimethyl-7-methylidenehexahydro-1H-spiro[Cyclopenta[*c*]pentalene-2,2'-[1,3]dioxan]-5(3*H*)-one (**12**):

Using 0.29 g of enone **11** as the substrate with 0.12 g (0.67 mmol) of 2-(trimethylsilylmethyl)allyl acetate, the method used to prepare compound (**10**) was employed to yield 0.14 g of ketal angular triquinane (**12**). 75% yield. ^{1}H NMR (600 MHz, $CDCl_3$) δ 4.75 (br s, 1H; H12), 4.66 (br s, 1H; H12), 3.55 (d, J = 3.8 Hz, 2H; O-CH_2), 3.48 (d, J = 3.8 Hz, 2H; O-CH_2), 3.05 (br m, 1H; H8), 2.97 – 2.78 (apparent td, 4H, H4α and β, H2α and β), 2.65 (m, 2H; H5, H7β), 2.08 (dd, 1H; H7α), 1.70 – 1.66 (br m, 3H; H9β, H11α and β), 1.34 (apparent t, J = 12.5 Hz, 1H; H9α), 1.04, 0.96 (S, S, 3H each; Me-16 and Me-17). ^{13}C NMR (151 MHz, $CDCl_3$ δ 208.8 (C6), 142.3 (C3), 134.7 (C12), 109.5 (C10), 72.7 (C13), 71.8 (C14), 42.4(C1, C8), 41.5 (C5), 41.4 (C7, C2), 37.1 (C4), 31.8 (C11), 30.2 (C15), 29.8 (C9), 22.5 (C16), 22.5 (C17). HRMS (EI-TOF): calculated for $C_{17}H_{24}O_3$ [M+] m/z 276.1725; observed m/z 276.1735.

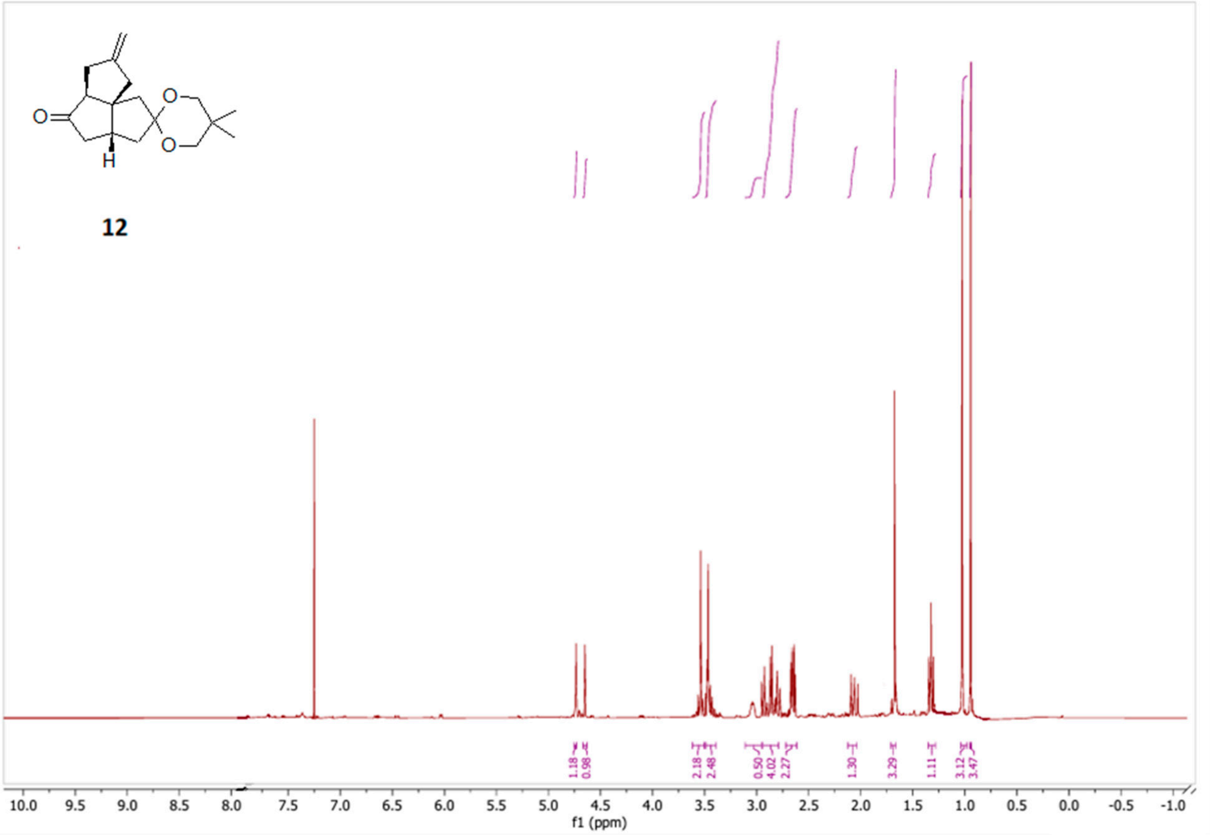

*Figure S19: ^{1}H NMR spectrum of angular triquinane **12** ($CDCl_3$).*

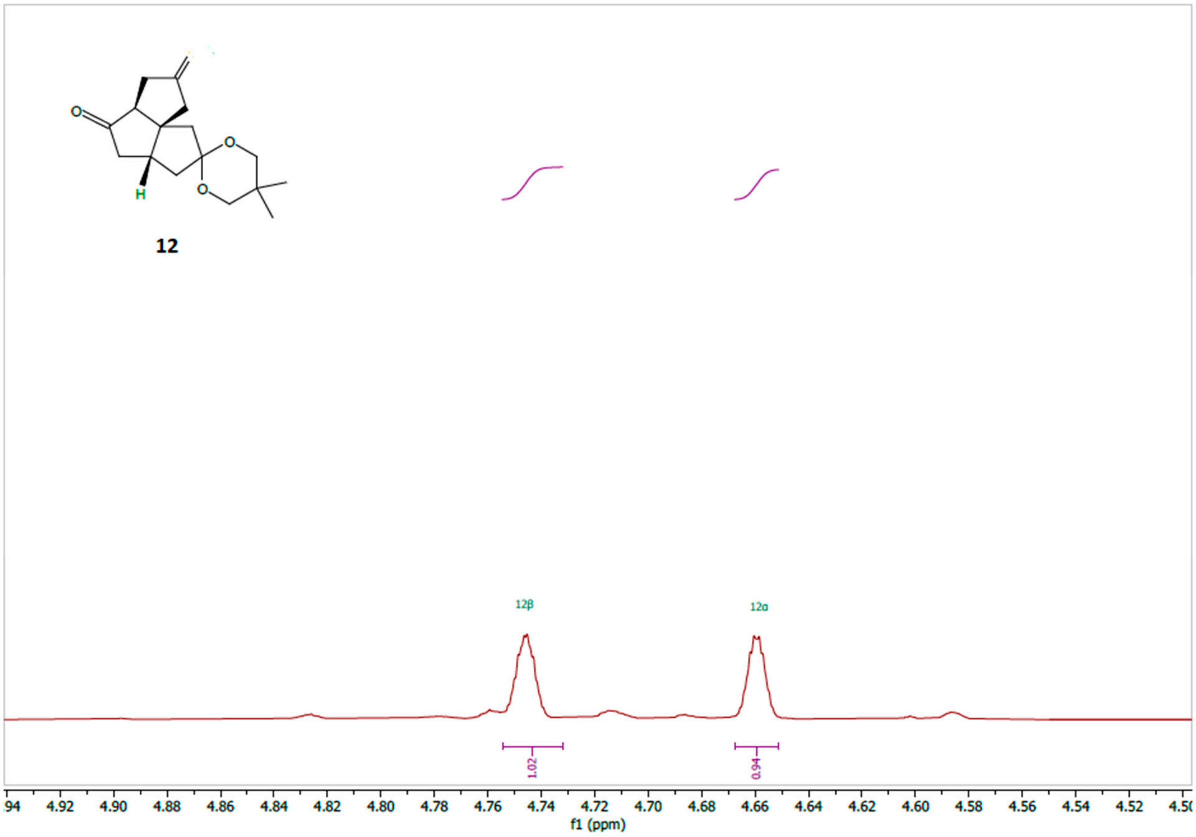

*Figure S20: ^{1}H NMR blowup of vinyl region of **12**.*

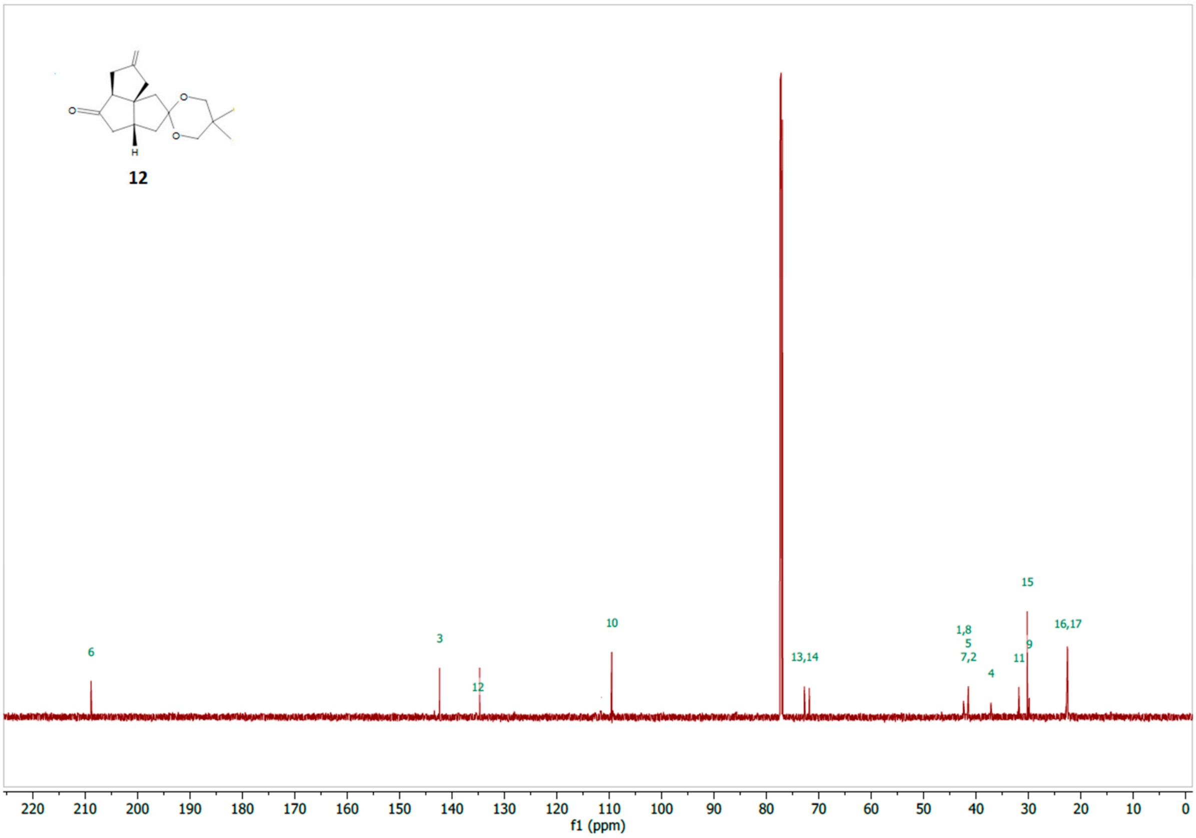

*Figure S21: ¹³C NMR spectrum of angular triquinane **12** ($CDCl_3$).*

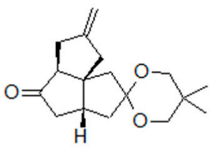

12

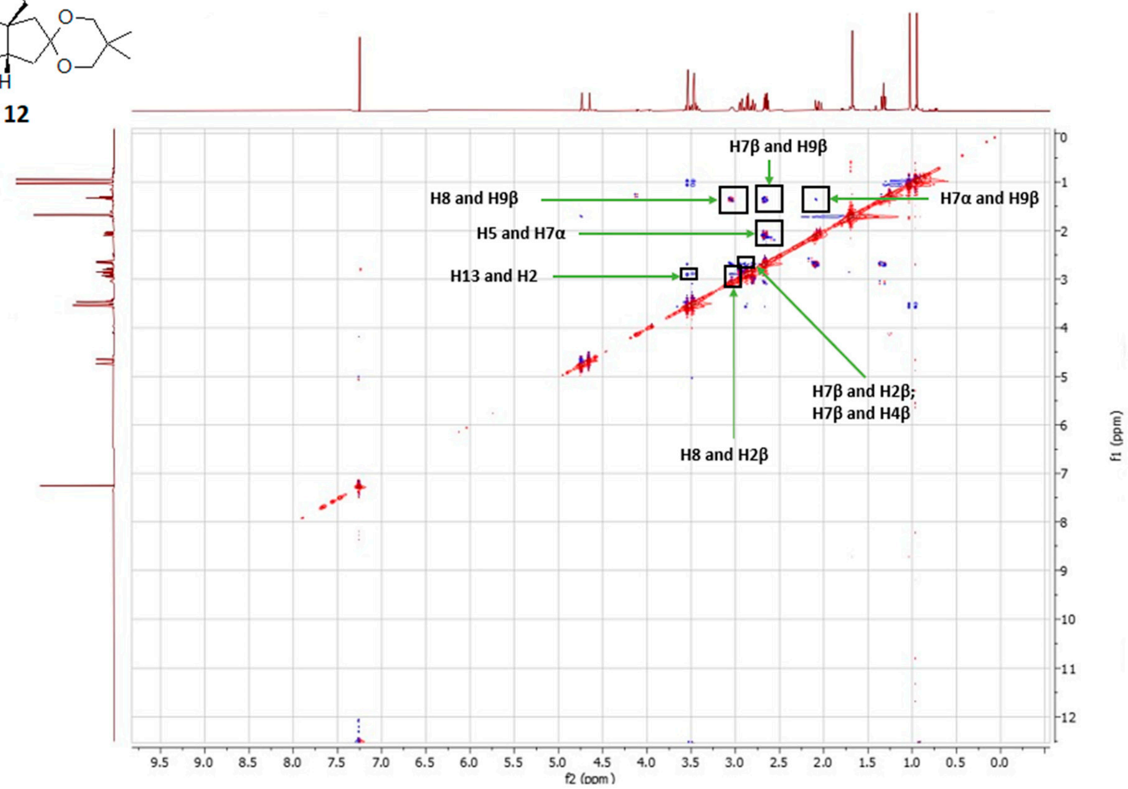

*Figure S22: NOESY spectrum of **12** ($CDCl_3$).*

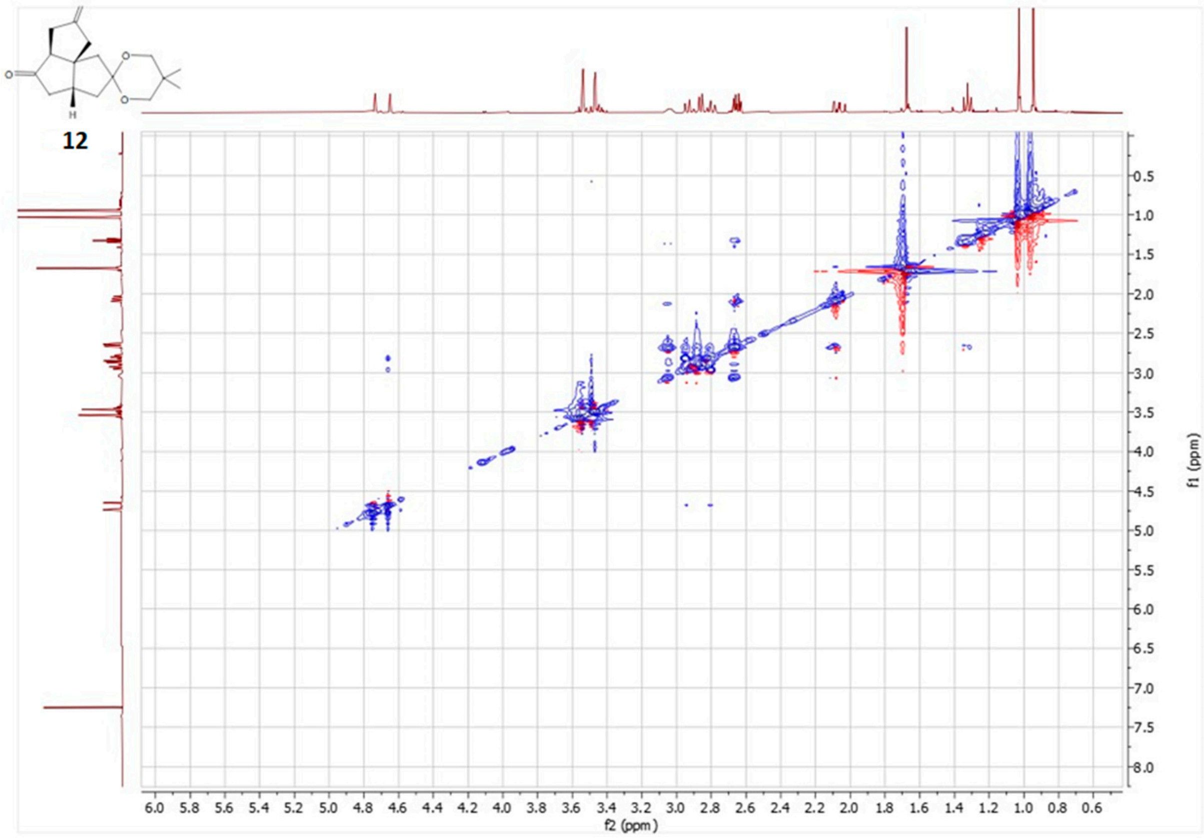

*Figure S23: TOCSY spectrum of **12** ($CDCl_3$)*
